# Editorial: Emotion regulation and cognitive processes

**DOI:** 10.3389/fnhum.2023.1250666

**Published:** 2023-07-20

**Authors:** Vilfredo De Pascalis, Carmen Moret-Tatay

**Affiliations:** ^1^Department of Psychology, Sapienza University of Rome, Rome, Italy; ^2^Facultad de Psicología, Universidad Católica de Valencia San Vicente Mártir, Valencia, Spain

**Keywords:** emotion, emotion regulation, cognition, neurophysiological techniques, multimodal approach

Emotion regulation is crucial in individuals' cognitive ability to manage and express their emotions effectively. This Research Topic delves into the neural processes and reactions associated with emotion regulation using various neurophysiological techniques, including electroencephalogram (EEG), event-related potentials (ERPs), functional magnetic resonance imaging (fMRI), and transcranial electrical stimulation (tES). Furthermore, it investigates the impact of different stimulus types, aesthetic preferences, and mindfulness practices on emotion regulation. The findings contribute to a comprehensive understanding of emotion regulation strategies and provide insights for potential therapeutic interventions.

Within this context, EEG and event-related potentials are valuable tools for investigating the neural processes and reactions related to emotion regulation. A piece of research (Yang et al.) has proposed distinct roles for the P100, N170, and P250 neural generators. These generators are involved in encoding comprehensive frames of reference, maintaining structural cohesion, and adapting to internal representations of emotional expressions.

In a study by Chen et al., the amplitude of the N100 (N1) component was examined using the mismatch negativity (MMN) in a group of young and middle-aged adults. They employed a modified oddball paradigm to compare responses to standard tones under fearful and neutral facial expressions. The results demonstrated that when exposed to fearful facial expressions, the amplitude of the N1 in response to classic techniques was smaller than in neutral facial expressions.

Additionally, the amplitude was more negative in young adults than middle-aged adults. These findings suggest that the perception of negative emotions visually facilitates the processing of auditory features and enhances acoustic change detection in middle-aged adults. However, this effect cannot compensate for the age-related decline in MMN amplitude.

Emotions are interconnected with our social interactions as we navigate the social realm by interpreting and choosing between conflicting emotional cues. The effective face-word Stroop (AFWS) is an experimental method that can help uncover the fundamental neural processes that support crucial social and emotional abilities. Regarding the timing, location, and sequence of control processes involved in responding to emotional conflicts during the AFWS, a study determined that conflict control processes occur within the components of Visually evoked potentials (Jamieson et al.).

On the other hand, the role of other internal variables has also been addressed. A study utilizing electromyography (Åsli and Øvervoll) discovered that the interaction between emotional expressions and model gender influences the magnitude of startle responses. Specifically, greater startle responses were observed when participants viewed angry faces displayed by male models and happy expressions displayed by female models. Another study investigating emotional stimuli in individuals with spinal cord injury (Pecchinenda et al.) proposed an integrated explanation for emotional conflict resolution during response activation tasks. This study emphasized the influence of emotional arousal and primed approach or avoidance motivation. Furthermore, they discussed the role of arousal in individuals with spinal cord lesions, providing insights into the typical mechanisms involved in emotional conflict control. Understanding emotional conflict control is crucial for adaptive behavior and may have implications for therapeutic interventions.

Fusina et al. conducted a study examining the Default Mode Network and Ventral Attention Network in individuals with high and low emotion dysregulation to explore the neurophysiological correlates of emotion dysregulation. The findings indicated high emotion dysregulation is associated with increased connectivity between the Ventral Attention Network and other networks, highlighting an intensified automatic attentional focus on internal states and emotionally intense thoughts in individuals prone to emotion dysregulation.

Using fMRI, a study by Fujiwara et al. demonstrated that individuals with a strong desire for praise exhibit lower activation in the inferior parietal sulcus during sincere praise, particularly after experiencing poor task performance. This finding suggests that negative feedback is suppressed to protect self-esteem. The study highlighted distinct neural patterns underlying the rewarding and socio-emotional effects of sincere praise compared to flattery.

The tES has gained popularity in modulating cognitive brain functions to improve neuropsychiatric conditions. A pilot study by Ghodratitoostani et al. collected data that informed the design of a well-controlled adaptive seamless Bayesian dose-response analysis. This study showed significant differences in self-reported tinnitus loudness before and during positive emotion induction.

Regarding other methodologies, Hu et al. suggested that the negative effect of emotional dissonance is influenced by work-family conflict. They conducted a moderated mediation model to explain the relationship between emotional leadership, emotional dissonance, and helping behavior. In another study, Lai et al. found that attachment style dimensions are associated with neural activation during the projection of mental states. Furthermore, Caprara et al. suggested that self-efficacy in managing positive emotions is associated with higher levels of chronic positive affect, lower negative affect, and greater life satisfaction, even after considering factors such as gender and age. The study also revealed that younger participants exhibit a stronger link between self-efficacy in using humor and life satisfaction compared to older individuals.

Focusing on the clinical population, results from a study suggested that individuals with Authentic pride (AP) demonstrated greater success in down-regulating negative emotions through cognitive reappraisal, both in daily life and experimental settings, compared to individuals with hubristic pride (Lin et al.). Moreover, a study conducted with patients suffering from mild traumatic brain injury examined negative frontal alpha asymmetry using EEG data and found greater right-sided frontal activity than healthy controls (Kuusinen et al.).

Regarding the type of stimuli used, Romeo et al. study reported that movies are more effective than slides in evoking emotions. The study also revealed that different brain regions are involved in processing emotions depending on the type of stimuli employed. Additionally, investigating the relationship between aesthetic preferences and neural responses contributes to establishing objective indicators for assessing aesthetic judgment and understanding the process of aesthetic cognition. Chen and Cheng conducted a study that provides valuable insights for quantitatively assessing aesthetics in various fields, such as environmental design, interior design, and marketing, mainly related to ceramic tiles. Differences in N100 and P200 amplitudes indicated that participants formed aesthetic perceptions of the tiles during the early and middle stages of vision, leading to varying levels of attention allocation. In the mid and late stages of visual processing, the disparity in LPP amplitude suggested that participants' impressions of the tiles were further deepened, forming a top-down emotion-driven evaluation.

Cognitive appraisal and associated emotional processes were also examined in motivational interviewing (Hui et al.). The amplitudes of the N400 brain wave component were correlated with participants' level of negative affect during the pre-contemplation stage. Another study revealed that post-error adjustments varied depending on the specific task context.

Mindfulness is another topic covered in this context. Despite the known benefits of mindfulness for mental health, drop-out rates among mindfulness practitioners can occur due to factors such as chronic stress, cognitive reactivity, and pathology. To address this issue, Sars proposes a framework called physical exercise (PE) augmented mindfulness, suggesting that regular physical exercise before meditation can enhance early-stage mindfulness. Neurocognitive research indicates that physical exercise (such as aerobic exercises or yoga) and mindfulness impact similar pathways involved in stress regulation and cognitive control, supporting the idea of synergistic effects between physical exercise and mindfulness. Studies focusing on the psychophysiological impact of physical exercise have shown that it can lead to short-term neurocognitive changes that facilitate cognitive control and the attainment of mindful awareness.

Lastly, in a review conducted by Küçüktaş and St. Jacques, the influence of visual perspective on the emotional aspects of autobiographical memory (AM) retrieval was examined. The review concluded that the impact of visual perspective depends on the emotional nature connected to the event.

By investigating the neural correlates and strategies associated with emotion regulation, this research paper aims to advance our understanding of how individuals skillfully handle and display their emotions in various contexts. The multimodal approach provides valuable insights into the underlying processes involved in emotion regulation and informs potential interventions for improving emotional wellbeing and adaptive behavior.

## Author contributions

All authors listed have made a substantial, direct, and intellectual contribution to the work and approved it for publication.

